# Transinguinal preperitoneal (TIPP) vs endoscopic total extraperitoneal (TEP) procedure in unilateral inguinal hernia repair: a randomized controlled trial

**DOI:** 10.1007/s10029-022-02651-5

**Published:** 2022-08-04

**Authors:** J. J. Posthuma, R. Sandkuyl, D.A. Sloothaak, A. Ottenhof, J. D. W. van der Bilt, J. A. H. Gooszen, P. C. M. Verbeek, K. H. in’t Hof

**Affiliations:** 1 Department of Surgery, Flevoziekenhuis, Hospitaalweg 1, 1315 RA Almere, The Netherlands; 2 Department of Surgery, Amsterdam UMC, Location AMC, Amsterdam, The Netherlands

**Keywords:** Inguinal hernia repair, Groin hernia, Chronic groin pain, TIPP, TEP, Lichtenstein

## Abstract

**Purpose:**

The Lichtenstein hernioplasty has long been seen as the gold standard for inguinal hernia repair. Unfortunately, this repair is often associated with chronic pain, up to 10–35%. Therefore, several new techniques have been developed, such as the transinguinal preperitoneal patch (TIPP) and the endoscopic total extraperitoneal (TEP) technique. Several studies showed beneficial results of the TIPP and TEP compared to the Lichtenstein hernioplasty; however, little is published on the outcome when comparing the TIPP and TEP procedures. This study aimed to evaluate outcomes after the TIPP vs the TEP technique for inguinal hernia repair.

**Methods:**

A single-center randomized controlled trial was carried out between 2015 and 2020. A total of 300 patients with unilateral inguinal hernia were enrolled and randomized to the TIPP- or TEP technique. Primary outcome was chronic pain (defined as any pain following the last 3 months) and quality of life, assessed with Carolinas comfort scale (CCS) at 12 months. Secondary outcomes were: wound infection, wound hypoesthesia, recurrence, readmission within 30 days, and reoperation.

**Results:**

A total of 300 patients were randomized (150 per group). After a follow-up of 12 months, we observed significantly less postoperative chronic groin pain, chronic pain at exertion, wound hypoesthesia, and wound infections after the TEP when compared to the TIPP procedure. No significant differences in quality of life, reoperations, recurrence rate, and readmission within 30 days were observed.

**Conclusion:**

We showed that the TEP has a favorable outcome compared to the TIPP procedure, leading to less postoperative pain and wound complications, whereas recurrence rates and reoperations were equal in both the groups.

## Introduction

Inguinal hernia repair is one of the most commonly performed surgical procedures worldwide with approximately 20 million cases every year [1, 2]. The open Lichtenstein tension-free hernioplasty is most commonly performed and recognized for its low recurrence rate and short learning curve, but associated with an undesirably high level of postoperative chronic groin pain, up to 10–35% [1, 3, 4]. Therefore, chronic groin pain is a common concern after inguinal hernia surgery, the condition assumed to be the result of entrapment, stretching, or damage to the ilioinguinal, iliohypogastric and/or genitofemoral nerve [5–10]. In the retroperitoneal space, the genitofemoral nerve follows the psoas muscle until dividing into the femoral and genital rami. The r. femoralis travels along with the psoas muscle underneath the inguinal ligament, where it innervates the skin of the upper leg. The r. genitalis travels along with the funiculus into the inguinal canal, where it innervates the cremaster and scrotal skin in men. In women, the genital branch accompanies the ligamentum rotundum, innervating the skin of the mons pubis and labia majora [11]. The ilioinguinal and iliohypogastric nerves arise as a single trunk from the lumbar plexus (Th12-L1), emerging along the upper lateral border of the psoas major and passes across the ventral surface of the quadratus lumborum muscle, where it divides into the iliohypogastric and ilioinguinal branches [11]. Here, the iliohypogastric and ilioinguinal nerves penetrate the transversus abdominis muscle to follow its course between the internal oblique muscle and external oblique muscle [12]. The iliohypogastric nerve finally pierces the external oblique aponeurosis just above the external inguinal ring, where it mainly provides cutaneous innervation of the medial thigh, pubic, and scrotal/labial area [12]. The ilioinguinal nerve becomes superficial by passing through the external inguinal ring anterior to the spermatic cord [13]. The sensory branches innervate the anterior 1/3 of the labium majus in females or the skin of the anterior 1/3 of the scrotum and root of the penis in males. Treatment of chronic groin pain can be challenging and may require several interventions including, local anesthesia/corticosteroids or additional surgery [14–16]. Several studies showed, that chronic groin pain might be dependent on the surgical technique, including identification and handling of inguinal nerves [16, 17]. Preperitoneal techniques have been developed to minimize these risks. The transinguinal preperitoneal patch (TIPP) and endoscopic totally extraperitoneal repair (TEP) are such procedures and previous studies showed advantages for both TEP and TIPP over the open Lichtenstein repair [17–19]. However, evidence on comparing TIPP and TEP directly is lacking. Therefore, we studied the difference in postoperative complications and quality of life (QoL) after inguinal hernia repair by means of TIPP or TEP in a single-center, randomized controlled trial.

## Methods

Between February 2015 and December 2020, patients with unilateral primary inguinal hernia were enrolled for participation and included patients were randomized, either for the TIPP or TEP. Patients younger than 18 years of age and patients with recurrent or bilateral hernias were excluded. Cases in which preperitoneal surgery had previously been performed were also excluded.

### Surgical procedures

The TIPP technique involves a standard anterior inguinal approach, with high dissection and preperitoneal reduction of the hernia sac through the internal ring. Blunt dissection of the preperitoneal space is carried out using one finger or large dissection gauze through the internal ring. Subsequently, the preperitoneal space is extended deep towards the epigastric vessels and transverse fascia in the direction of the pubic tubercle. The hernia patch is introduced in the preperitoneal space through the internal orifice. External oblique aponeurosis repair was performed superficial to the spermatic cord to restore normal anatomy. In this study, we used a 16 × 9.5 cm Polysoft™ BARD Hernia Patch with memory ring (BARD Benelux, Belgium).

The TEP technique is performed using a subumbilical port for retromuscular and preperitoneal access. Carbon dioxide is insufflated to a pressure of 10 mmHg. Two 5-mm trocars are placed in the midline or one trocar in the midline and the other laterally in Bogros’ space. The hernia sac was reduced and the peritoneum mobilized to expose the triangle of doom (bounded by: vas deferens medially, spermatic vessels laterally, and peritoneal fold dorsally) and laterally to the Bogros’ space. An unfixed non-absorbable 15 × 12 cm mesh (Polypropylene, Prolene, Ethicon, NJ, USA) was placed to cover the inguinal and femoral areas.

### Outcome

Primary outcome was chronic pain as assessed with the VAS score (0–100, with 0 indicating no pain and 100 indicating the worst possible pain) and defined as any pain following the last 3 months. Pain was classified as mild pain (VAS 10–30), moderate (VAS 40–60), or severe (VAS > 60) [20]. Quality of life was measured by the Carolina comfort scale (CCS) at 12 months postoperatively [21]. The CCS is a questionnaire that quantifies the severity of pain, foreign body sensation, and movement limitation from the hernia of the surgical site during the following 8 activities: lying down, bending over, sitting up, activities of daily living, coughing or deep breathing, walking, climbing stairs, and exercise. The answers are recorded on a 6-point Likert scale, which ranges from an absence of symptoms (score 0) to disabling symptoms (score 10). Questionnaires were completed preoperatively and postoperatively at 12 months [21]. Hereto, chronic pain at exertion is defined as experiencing pain every time during one or more of the activities reported in the Carolina’s comfort scale during at least 3 months. Secondary outcomes were: wound infection, readmission within 30 days, recurrence, and procedure required reoperation, the latter obtained from a questionnaire that was sent to all patients. In addition, wound hypoesthesia was defined as, diminished or reduced perception of mechanical stimuli in the previously operated region. Hereto, the mechanical stimuli were tested by touch and pressure. If one or both tests were aberrant, this was scored a wound hypoesthesia.

### Sample size and randomization

The sample size was based on the hypothesis that 5% of the patients having TEP [22] and 10% having TIPP [4] procedure would experience pain at 1 year, defined as any pain during the last 3 months. A total of 100 patients in each group were required to detect a difference (*α* = 0⋅05, power of 90%). With an anticipated dropout rate of 33%, 150 patients were to be included in each group. The allocation ratio was 1:1. The randomization sequence used for the allocation of patients was created. The randomization took place when the patient was scheduled for operation. The patient was informed of the allocated procedure and surgeons were informed about the study and the enrollment of patients.

### Statistical analysis

Statistical calculations were done with IBM SPSS^®^ Statistics version 25 (IBM, Armonk, New York, USA). Mean and standard deviation was given in evenly distributed results, whereas median and interquartile range (IQR) was given in unevenly distributed results. Categorical variables were analyzed using Fischer’s exact test (2-sided). The absolute risk reduction (ARR) is the arithmetic difference between the rates in the TIPP vs TEP procedure, thereby calculated by: ARR = event rate in the TIPP group − event rate in the TEP group. The number needed to treat (NNT) is the number of patients one needs to treat to prevent one additional chronic inguinal pain patient. The NNT is calculated by 1/ARR. A *p* value < 0.05 was considered statistically significant.

The trial was approved by the Regional Ethical Review Board in Amsterdam, The Netherlands, and informed consent was signed by all the patients.

## Results

**Fig. 1:**
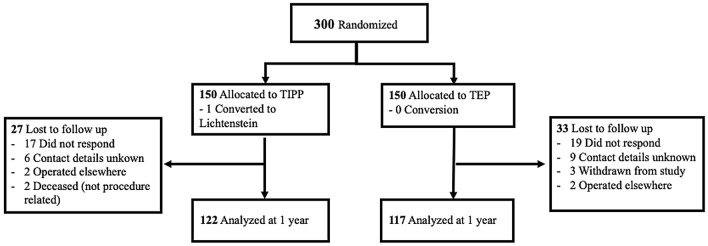
Flowchart randomization and follow-up

A total of 300 patients were included, 150 for the TIPP and 150 for the TEP procedure. At 1 year, 80% of the patients responded to the questionnaire, leaving 122 patients in the TIPP group and 117 patients in the TEP group (Fig. 1). Preoperative data are presented in Table 1.

**Table 1:** Baseline characteristics for patients undergoing inguinal hernia repair

	TIPP (*n* = 122)	TEP (*n* = 117)
Age, mean (SD)	61.4 (10.2)	59.8 (9.8)
Gender (% male)	95.9%	97.4%
ASA classification		
I (%)	52 (42.7%)	44 (37.6%)
II (%)	63 (51.6%)	65 (55.6%)
III (%)	7 (5.7%)	8 (6.8%)
IV (%)	0 (0%)	0 (0%)
Pain sensation at rest pre-operative (VAS) (median (IQR)	36 (25–46)	32 (23–44)
Type of anesthesia, *n* (%)		
General	84 (69%)	117 (100%)
Spinal	38 (31%)	0 (0%)
Local	0 (0%)	0 (0%)

*SD* standard deviation, *ASA* American society of anesthesiology, *VAS* visual analogue scale

### Postoperative chronic groin pain

**Fig. 2:**
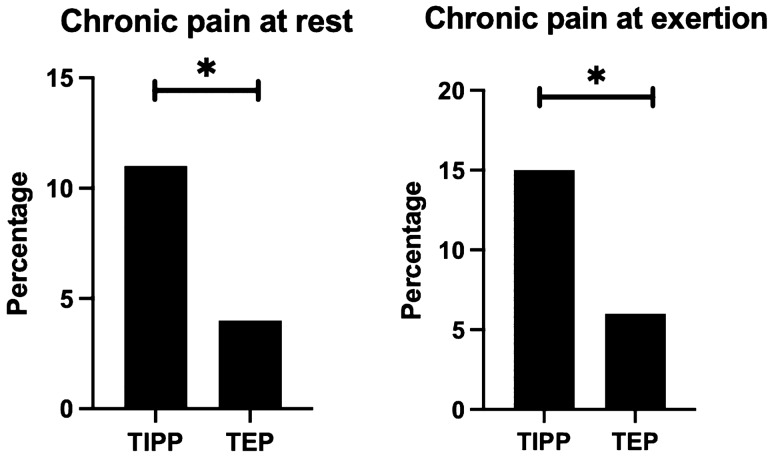
Postoperative chronic pain at rest and during exertion 1 year after inguinal hernia repair by TIPP or TEP procedure. *Statistical difference between two groups

**Fig. 3:**
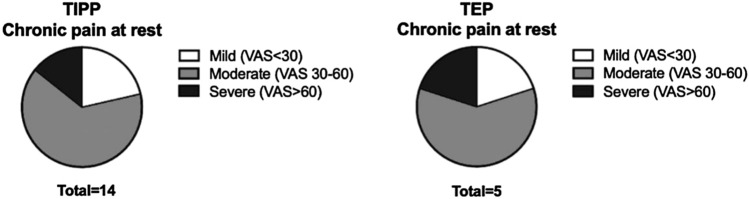
Distribution of pain scores after inguinal hernia repair by TIPP vs TEP

After 1 year, significantly lower postoperative chronic groin pain at rest was observed after the TEP when compared to the TIPP (4.3% vs 11.4%, *p* < 0.05) (Fig. 2). More in detail, mild pain was present in 0.9% of patients after the TEP and 2.5% after the TIPP, moderate pain in 2.5% after the TEP procedure and 7.3% after the TIPP procedure, and severe pain (VAS > 60) was found in 0.9% after the TEP procedure and 1.6% after TIPP procedures (Fig. 2). Furthermore, the TEP procedure was associated with less chronic groin pain during exertion in relation to the TIPP procedure (6.0% vs 14.8%, *p* < 0.01) (Fig. 3).

### Quality of life

Besides chronic pain, no statistical differences were found in quality of life between the TEP and TIPP after 12 months. Overall, mesh sensation was similar (TIPP: 11% vs TEP: 9%, *p* = 0.63) and also movement limitations were not statistically different between the TIPP and TEP (6% vs 4%, *p* = 0.67).

### Wound complication

Fewer patients experienced wound hypoesthesia after TEP when compared to the TIPP (0.9% vs 9.8%, *p* < 0.01). Also, lesser wound infections were seen after the TEP (0% vs 4.1%, *p* < 0.05), where infections were mainly caused by superficial wound infections (TEP: 0%, TIPP: 3.3%) and deep wound infections were rare (TEP 0% vs TIPP 0.8%) (Table 2).

**Table 2:** Clinical outcome 1 year after inguinal hernia repair by TIPP or TEP

	TIPP (*n* = 122)	TEP (*n* = 117)	*p* value	ARR	NNT (1/ARR)
Chronic pain at rest (VAS > 0)	14 (11.4%)	5 (4.3%)	0.03*	7.1%	14
Mild (VAS < 20)	3 (2.5%)	1 (0.9%)	0.62	–	–
Moderate (VAS 30–60)	9 (7.3%)	3 (2.5%)	0.13	–	–
Severe (VAS > 60)	2 (1.6%)	1 (0.9%)	0.99	–	–
Chronic pain at exertion (VAS > 0)	18 (14.8%)	7 (6.0%)	0.02*	8.8%	11
Mild (VAS < 20)	4 (3.2%)	1 (0.9%)	0.37	–	–
Moderate (VAS 30–60)	10 (8.4%)	4 (3.4%)	0.16	–	–
Severe (VAS > 60)	4 (3.2%)	2 (1.7%)	0.68	–	–
Discomfort in daily life	9 (7.4%)	4 (3.4%)	0.14	–	–
Wound hypoesthesia					
Reduced or diminished sensation to touch or pressure in operated region	12 (9.8%)	1 (0.9%)	0.001*	8.9%	11
Wound infection	5 (4.1%)	0 (0%)	0.03*	4.2%	24
Superficial	4 (3.3%)	0 (0%)	0.12	–	–
Deep	1 (0.8%)	0 (0%)	0.99	–	–
Recurrence	6 (4.9%)	2 (1.7%)	0.28	–	–
Readmission within 30 days	1 (0.8%)	0 (0%)	0.5	–	–
Reoperation	5 (4.0%)	3 (2.6%)	0.38	–	–
For recurrence	2 (1.6%)	2 (1.7%)	0.99	–	–
For infection	2 (1.6%)	0 (0%)	0.49	–	–
For chronic groin pain	1 (0.8%)	1 (0.9%)	0.99	–	–
Follow-up, months, mean (SD	12.4 (4.3)	12.8 (4.8)		–	–

*ARR* absolute risk reduction, *NNT* number needed to treat, *VAS* visual analogue scale, *SD* standard deviation

*Statistically significant difference between TIPP vs TEP

### Recurrence and reoperation

**Fig. 4:**
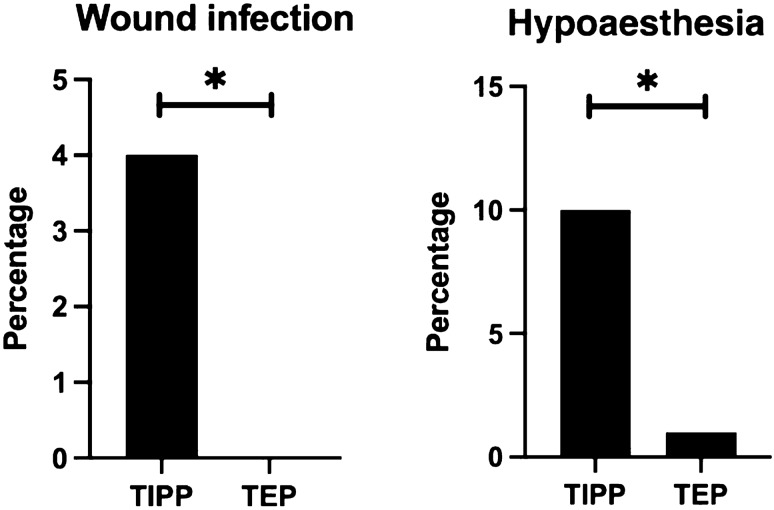
Wound complications after TIPP vs TEP procedure. *Statistical difference between the two groups

After an average of 12 months, recurrence was noted in two patients in the TEP group (1.7%) and six patients after the TIPP procedure (4.9%, *p* = 0.28). The latter, leading to additional operative treatment in two patients for both groups (1.7% and 1.6%). In total, three patients in the TEP group and five patients in the TIPP procedure went for reoperation (2.6% vs 4.0%, *p* = 0.38) (Fig. 4), and reasons for reoperations are presented in Table 2.

## Discussion

This study indicates that patients with inguinal hernias have less chronic groin pain after the TEP procedure than after the TIPP. The difference in the proportion of postoperative chronic groin pain in the two groups appears to be clinically important, given the effects on QoL and the need for additional treatments [23–27].

It has previously been thought that nerves are protected from contact with mesh during preperitoneal procedures; however, a Mesh placed in the preperitoneal space might abut the genital branch of the genitofemoral nerve, depending on the exact location of the prosthesis [28]. Chen et al. outlined that a membranous layer of the extraperitoneal fascia exists, dividing the preperitoneal space into a visceral and parietal compartment [29]. The genitofemoral nerve is located in the parietal compartment and protected by a membranous layer dividing the visceral and parietal compartments. In this regard, placement of the mesh in the visceral compartment, (as is done during a TEP procedure), is theoretically less likely to cause genitofemoral neuralgia when compared to, unintentionally, placement of the mesh in the parietal compartment. In this regard, an important anatomic consideration is that the preperitoneal dissection is functionally blind during the TIPP procedure, therefore more prone to malpositioning of the mesh in the parietal compartment. This is consistent with the finding that the blind dissection during the TIPP procedure was associated with a low number of nerve identifications [4, 28]. During the TEP procedure, however, all structures can be visualized including nerves and vascular structures as well as the separation between the visceral and parietal compartments. The latter and the former might explain the difference in chronic groin pain after the TIPP and TEP procedure.

In addition to this, we hypothesize that the difference in approach between the TIPP and TEP might explain the difference in postoperative chronic pain after the TIPP vs TEP. The approach of the TEP procedure involves a subumbilical incision and two incisions placed in the midline or one in the midline and the other laterally in Bogros’ space, while the open anterior inguinal approach during the TIPP procedure provides access to the internal inguinal ring by dissecting through the abdominal wall. Given the anatomical location of the ilioinguinal and iliohypogastric nerves in the abdominal wall at this region, we consider that the ilioinguinal and iliohypogastric nerves are at risk for unintentional damage during the open anterior approach and unlikely during the TEP approach [11, 13].

In our study, we observed no statistically significant differences in recurrence rates. This was in line with other studies, where the specific operation technique does not influence recurrence rates [22, 30, 31], and others found that the recurrence rate depends on the surgeon’s experience [32]. Given the long learning curve in the TEP procedure, this seems an important factor in evaluating recurrence rates after TEP procedures. In our center, inguinal hernia repairs are performed by experienced surgeons, or performed by a resident under strict supervision of an experienced surgeon.

## Conclusion

This randomized controlled trial revealed significantly better results in postoperative chronic pain after 12 months for the TEP procedure as compared to the TIPP procedure for inguinal hernia repair, whereas we observed no significant differences in: recurrence, readmission within 30 days, or reoperation.
